# Methodological Considerations for Setting Up Deep Brain Stimulation Studies for New Indications

**DOI:** 10.3390/jcm11030696

**Published:** 2022-01-28

**Authors:** Jana V. P. Devos, Yasin Temel, Linda Ackermans, Veerle Visser-Vandewalle, Oezguer A. Onur, Koen Schruers, Jasper Smit, Marcus L. F. Janssen

**Affiliations:** 1 School for Mental Health and Neuroscience, Maastricht University, 6229 ER Maastricht, The Netherlands; linda.ackermans@mumc.nl (L.A.); ja.smit@zuyderland.nl (J.S.); m.janssen@maastrichtuniversity.nl (M.L.F.J.); 2 Department of Ear, Nose, Throat, Head and Neck Surgery, Maastricht University Medical Center, Maastricht University, 6229 HX Maastricht, The Netherlands; 3 Department of Neurosurgery, Maastricht University Medical Center, Maastricht University, 6229 HX Maastricht, The Netherlands; 4 Department of Stereotactic and Functional Neurosurgery, Faculty of Medicine and University Hospital Cologne, University of Cologne, 50923 Cologne, Germany; veerle.visser-vandewalle@uk-koeln.de; 5 Department of Neurology, Faculty of Medicine and University Hospital Cologne, University of Cologne, 50923 Cologne, Germany; oezguer.onur@uk-koeln.de; 6 Department of Psychiatry and Neuropsychology, Maastricht University Medical Center, Maastricht University, 6229 HX Maastricht, The Netherlands; koen.schruers@maastrichtuniversity.nl; 7 Department of Ear, Nose, Throat, Head and Neck Surgery, Zuyderland Medical Center, 6419 PC Heerlen, The Netherlands; 8 Department of Clinical Neurophysiology, Maastricht University Medical Center, Maastricht University, 6229 HX Maastricht, The Netherlands

**Keywords:** deep brain stimulation, first-in-human, methodology, feasibility, safety

## Abstract

Deep brain stimulation (DBS) is a neurosurgical treatment with a growing range of indications. The number of clinical studies is expanding because of DBS for new indications and efforts to improve DBS for existing indications. To date, various methods have been used to perform DBS studies. Designing a clinical intervention study with active implantable medical devices has specific challenges while expanding patient treatment. This paper provides an overview of the key aspects that are essential for setting up a DBS study.

## 1. Introduction

**Figure 1:**
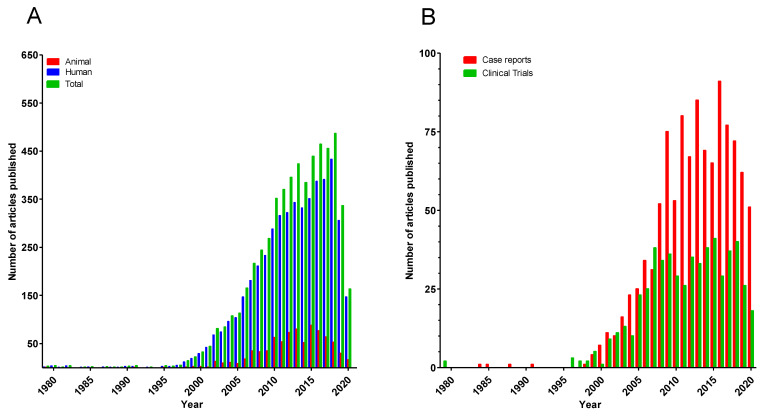
Result of PubMed searches of (**A**) number of articles published related to “deep brain stimulation (DBS)” in animal studies (red), human studies (blue), and both (green) and (**B**) number of case reports on DBS (red) and clinical trials (as defined by the PubMed filters) (green).

As reflected by the growing number of publications (Figure 1A), DBS is being investigated for an increasing number of disorders [1]. Interestingly, randomized trials are still outnumbered by case reports (see Figure 1B) on headaches [2], addiction [3,4], obesity [5], eating disorders [6], and stroke recovery [7]. Published case reports show high variation in methodology, making comparisons difficult. There is an additional risk of publication bias because often, only cases with positive outcomes are published [8]. Clinical trials also vary in methodological aspects [9,10].

The variation in methodology in clinical DBS studies can complicate decisions when setting up a clinical study. This review focuses on the methodological designs of DBS studies and discusses challenges and possibilities. With this review, we aim for more homogenous strategies in the design and preparation of future DBS trials. 

## 2. Phases in Setting Up a Trial

When testing a new pharmacological substance, the dosage, side effects, and pharmacological properties are considered in strict phases during a clinical trial (see Table 1) in order to gradually test the benefits for humans [11]. Throughout these phases, the substance itself remains the same, and the main goal is to test its safety for human use.

When testing a new medical device, these guidelines are largely lacking. Similar to pharmacological trials, phases can be adopted in the assessment of an active implantable medical device (AIMD) for a new indication. The safety and biocompatibility of DBS devices have already been proven, and they are being used in clinical practice. FDA approval and/or CE mark are available for DBS treatment for specific disorders targeting listed brain regions. A trial to test the treatment of a new indication using DBS is similar to testing a pharmacological substance, as it uses an existing and approved AIMD. The device, similar to a substance, will not change during the trial. It is used as it would be in standard practice but applied to a new disorder (albeit in a different brain region). In pharmacological trials, the dosage and duration of treatment vary; in DBS, the stimulation protocol is adapted to acquire the best treatment outcomes. 

An outline of the model for DBS for new indications analogous to pharmaceutical clinical trial phases is suggested in Table 1 [12]. In the following sections, we further suggest how to structure the preclinical phase and phase 1 trials for a new DBS indication.

## 3. Preclinical Phase

When DBS is expected to alleviate a specific neurologic or psychiatric disorder, a preclinical phase should first be initiated to provide sufficient evidence prior to conducting a first-in-human study. 

### 3.1. Discovery and First Evidence 

Advances in the field of neuroscience, such as insights into the neurophysiology of particular brain areas, brain circuits, and disorders, may spark new interest in DBS as a viable treatment option. Some beneficial side effects may be observed in patients treated with DBS for a preapproved indication, for example, the suppression of alcohol dependence [3], the success of smoking cessation [1], and the alleviation of tinnitus [13]. While these findings are valuable medical observations, it is unethical to apply them immediately in clinical practice without prior research. An initial investigational step is to research whether a side effect that occurred in a small group of patients can be extrapolated. This can be completed through a literature review or an active investigation of the phenomenon with a retrospective study [14]. Before application in humans is considered, we advocate to first collect preclinical evidence in both animals and humans.

### 3.2. Evidence in Humans

In humans, indications to apply DBS for a specific disorder in a certain brain region can be obtained by collecting evidence provided by different scientific methods. Sources of information include clinical reports from intended and unintended lesions, knowledge obtained from imaging studies, and neurophysiological studies utilizing electroencephalography (EEG) and magnetoencephalography (MEG). 

Disorders responsive to ablative surgery will likely benefit from DBS because of similar clinical effects [15,16]. In the future, evaluation on an individual basis using the non-ablative functionality of focused ultrasound is potentially a promising predictive tool [17]. Additional information can be collected by identifying a disorder’s specific neuronal correlate using both invasive and noninvasive neurophysiological methods. 

Non-invasive measurements, such as magnetic resonance imaging (MRI) and positron emission tomography (PET), give insight into functional and structural properties of neuronal areas, as were performed prior to the application of DBS for cluster headaches [18]. Additionally, the reversal of disorder-specific activity patterns after successful treatment can be demonstrated, e.g., treatment-resistant depression where target selection was based on activity alterations after successful pharmacological treatment [19]. 

EEG and MEG allow for the acquisition of neuronal activity in the (sub-)cortical regions. Using event-related evoked potentials, the response of healthy participants presented with stimuli is observed. By comparing these responses to ones from patients with certain disorders, it is possible to identify divergent disorder-related signals [20]. In line with this, non-invasive methods, such as transcranial magnet stimulation (TMS) and transcranial direct current stimulation (tDCS) combined with fMRI or electrophysiological studies, show potential to predict the outcome of DBS in a preclinical phase. 

Invasive measurements in humans, such as local field potentials (LFP), can only be performed in patients who undergo a neurosurgical procedure (such as DBS or stereo-EEG). Consequently, data from healthy controls are lacking. LFPs reflect the coherent dendritic activity of the surrounding area and can reveal subtle and specific interactions in local cell assemblies [21]. One use of this in a preclinical phase is when patients undergo DBS for an approved disorder and the DBS electrode traverses the area of interest on its way to the target. It is possible to briefly record from this area or even stimulate to observe the effect of stimulation using short assessment scales or tasks. The latter has been performed in patients who were treated for Parkinson’s disease (PD) and had concurrent tinnitus. The electrode traversed the caudate nucleus, and the effect of stimulation was tested [22]. Today, LFP recordings can also be obtained chronically since internal pulse generators (Percept PC Medtronic, Minneapolis, MN, USA, and AlphaDBS System, Newronika, Milan, Italy) are able to record [23,24]. The advantage is that these LFP recordings can be conducted while the patient is in a non-clinical setting, such as their home. During chronic recordings, fluctuations related to medication or daily activities can also be observed. It should, however, be noted that these recordings may suffer from electrical artifacts that disturb the signal [24]. Recently, multi-day intracranial electrophysiology was used to identify a biomarker for depressive symptoms in an individual and identify a brain region to target in surgery. Subsequently, a chronic deep brain sensing and stimulation device was implanted to resolve depressive symptoms [25]. However, this innovative approach needs further validation. 

### 3.3. Evidence in Animals

Preclinical studies can provide information to increase our scientific knowledge of neural circuitry. Such information can be used to identify potential new DBS targets and novel stimulation paradigms without subjecting patients to significant risks [26,27]. In addition, targets suggested through these methods can first be stimulated in animals to estimate the effects and screen for potential side effects. An example of an indication that was first noticed as a side effect of DBS is tinnitus alleviation [28]. A retrospective questionnaire study showed that this was more than a coincidental finding [13]. A rat model was used to test the safety and feasibility of three potential targets [29,30,31] before moving toward a first-in-human implantation. For this reason, an important addition to current DBS research was the development of robust translational models [32]. 

Currently, a growing number of psychiatric disorders are being considered for DBS. Psychiatric disorders are often multifactorial, involving both social and cultural aspects, and can therefore be challenging to study in animal models. Still, animal models can be used to study symptom reduction by DBS from a biological perspective [26,33]. Because of the complex nature of psychiatric disorders, it has been suggested to focus on the main symptoms of a psychiatric disorder that need treatment rather than resolving all aspects of the complex and multifaceted disorder [34]. Conversely, similar studies have provided evidence to rule out specific brain areas as suitable DBS targets [35]. 

### 3.4. Selection of the Target Brain Area

A thorough investigation of the most optimal brain region(s) should be considered without exception, even when a single case reports a beneficial effect on a specific symptom or disorder. In such a case, it is tempting to use the target that was used when the beneficial side effect first occurred. This target is often a well-studied area, which makes it easier to obtain approval from the responsible authorities and DBS manufacturer prior to performing a first-in-human study. However, in the preclinical phase, it is important to also consider other potential targets using findings from both human and animal research. 

When choosing a target, it can be helpful to consider potential stimulation-related side effects. First assumptions can be made using topographic and functional information of the surrounding structures. Lessons can also be learned from both intentional and unintentional lesions in patients, for example, those caused by a stroke [22]. In addition, it is important to assess the trajectory of the DBS lead to avoid passage through the ventricles, vulnerable brain regions, and blood vessels within the trajectory. It has been shown that the trajectory of the DBS lead can significantly affect the outcome of the surgery [36,37,38] and should thus be considered carefully. For targeting, high-resolution (at least 3T) MRI should be available, including a sequence with gadolinium to show the brain vessels. Using different sequences, specific brain regions may be directly visualized, or tracts can be visualized using diffusion tensor imaging. Direct targeting is preferred over indirect targeting using standard coordinates from brain atlases. Additionally, depending on the target, MER can be used to define the dorsal and ventral borders of the intended target. 

## 4. Setting Up a Phase 1 Trial 

When sufficient evidence is gathered in favor of DBS being a valid and safe treatment for a disorder, a first-in-human trial can be designed. The knowledge from the preclinical phase, the optimal target and parameters, the expected effect, and all potential side effects need to be taken into account during this design. 

In order to make information on trials that are being conducted more readily available, a database of trials should be created. Registering trials before conducting them should be part of good clinical practice and should be required as a condition for publication. A uniform registration system for case studies and series for relatively rare indications would also be useful. This would enable clinicians to easily retrieve information on previous and ongoing studies. These registers would provide better insight into DBS targets and stimulation paradigms tested in order to design new studies and prevent replication of negative studies, thereby preventing patients from being exposed to avoidable risks. 

### 4.1. Patient Screening and Selection 

A larger increase in patients treated for new indications compared to ones treated for approved indications was shown between 2002 and 2011. The patients treated for new indications were younger with lower comorbidity scores, which is often a consequence of more restrictive patient selection in research [39]. When DBS is applied to treat a new disorder, novel selection criteria need to be developed [40,41] (see Table 2). In general, DBS surgery is performed when patients suffer severely and persistently from a specific disorder. Even in standard care, DBS is an elective procedure aimed at treating severe symptoms that are refractory to other less invasive treatment options. For example, it is estimated that in the US, only 3% of all PD patients are referred for DBS surgery [42]. In any case, DBS does not cure the patient of the disease; it merely treats debilitating symptoms. In a trial, therefore, patients should be refractory to standard medical care, such as pharmaceutical and/or behavioral therapy [43]. It may be necessary to include a measure to establish the severity of the disorder being investigated and set a cut-off score for homogeneous selection [44]. Although a strict cut-off score may aid decision-making, the main goal of the patient selection criteria should be to optimize the individual risk–benefit ratio. Inclusion criteria should aim to describe which patients are likely to benefit most from the intervention. By contrast, exclusion criteria can describe which patients are not likely to benefit from the treatment [45] or for whom the risks outweigh the potential benefits.

Ideally, patients should have no serious medical comorbidities that could compromise DBS benefits or amplify surgical risk [43,46]. It is also preferred that patients have no cognitive deficits and no disabling or untreated behavioral, psychiatric, or mood deficits unless these are a result of the disorder being treated [41,46]. It is almost impossible to have patients without comorbidities, especially in a refractory population; therefore, a balance should be struck between the risks and benefits for each patient. In sum, patients with comorbidities that increase the risk of the procedure should be excluded. When comorbidities preclude full participation in the procedure or postoperative care or compromise the accurate assessment of the outcomes, inclusion should be discussed with the multidisciplinary team and the patient [47,48]. 

Within the selection procedure, an MRI scan is indispensable for locating the target area. Additionally, it helps to exclude patients with structural lesions or anatomical abnormalities [49]. Because of the necessity to conduct a preoperative MRI, exclusion should be considered in case of a contraindication for MRI or when no previous MRI data are available.

A pre-operative neuropsychological evaluation consists of a battery of neuropsychological assessments. Content may vary depending on the indication, including measures of intelligence, tests of cognitive functioning, mood, and quality of life [50,51]. If considered relevant or judged to be important by the neuropsychologist, it is possible to evaluate personality, coping responses, and stressors [51]. This screening is performed to exclude patients with significant pre-existing neuropsychological abnormalities in comparison to the general patient population. In addition, it is important to have a preoperative baseline measure of cognitive functioning in order to determine whether potential postoperative changes in cognitive functioning are related to the DBS [51,52].

In refractory patients, depression and anxiety are highly prevalent. Severe anxiety can be a major obstacle for awake stereotactic surgery, and depression can severely affect pre- and postoperative measures. If severe anxiety or depression is present and it is not a symptom of the investigated disorder, the patient should be counseled and treated before surgery, or if needed, excluded from the study [53]. In any case, good general and mental health, while keeping in mind the indication, is important for experimental DBS treatment. This is even more relevant if the electrodes are implanted under local anesthesia and the patient needs to be awake in order to interact during the procedure [54]. Depending on the circumstances, general anesthesia or procedural sedation and analgesia can be opted for to safeguard the patient’s comfort. 

Age has not been established as an important predictor for postoperative benefit following DBS procedures [49]. It does, however, contribute to the way patients cope with the surgical procedure and how they behave postoperatively. Younger patients usually recover faster and experience little or no cognitive dysfunction [53,55]. In comparison, older patients are more prone to experience short-term cognitive dysfunction (delirium), which can last up to a week after surgery [53,55]. Since calendar age does not necessarily correlate with biological age, this should be judged on an individual level, taking into account medical comorbidities, mental health, and physical functioning [53]. For these reasons, no specific cut-off score for age can be proposed. Important factors to take into account when considering age are the cultural, ethical, and legal implications concerning informed consent in children. The legal regulations in children vary between countries (when participants are under the age of 18) and should, in all cases, be fully adhered to. We believe that the involvement of family, guardians, and caregivers is important for all participants, and even more so in young participants. Additionally, this may even be legally necessary in order to obtain valid informed consent. 

In all cases, it is important to have an in-depth conversation with patients and their families or caregivers concerning their knowledge and expectations of what the study procedure entails, including both surgery and the postoperative period. Creating realistic expectations of outcomes for both the patient and the family [53], as well as determining the extent to which support (physical and emotional) is available to the patient [46], can be challenging. Patients must also be prepared for the possibility that DBS will not work or may have a negative impact on their quality of life. They should have the personal resilience and social support to live with possible negative outcomes [47]. Another important topic to discuss is the patient’s willingness to agree to multiple programming adjustments, randomizations, and the recording of outcome measures, which represent significant time commitments. The patient and their family must be motivated to participate in all required evaluations and adjustment procedures. DBS in an experimental setting should only be offered to a patient who has a good understanding and realistic expectations [46]. 

In practice, patients with absolute contraindications (e.g., severe medical comorbidities) should be excluded. Relative contraindications should be discussed within the multidisciplinary DBS team (e.g., diabetes or psychiatric diseases that are under control). Other factors, such as social circumstances, financial issues, availability for follow-up, and personal expectations, need to be evaluated on an individual basis. For example, a patient should be able to visit the hospital frequently because fine-tuning of stimulation parameters is essential to the outcome [49]. After a thorough evaluation by each of the specialists, patients should be selected in a multidisciplinary meeting [41,49].

Finally, it is advisable to involve patient associations. They have cooperative talents, including a good understanding of the needs of patients, and can comment on the feasibility of research expectations.

### 4.2. Power Calculation

The main goal of a first-in-human phase 1 trial is threefold: (1) to evaluate safety of the selected target, (2) to determine safe/optimal stimulation parameters, and (3) to identify side effects. These small trials show feasibility and may provide the first proof of effectiveness for future research. These results should be used to design sufficiently powered studies in the next phases. We encourage consulting a statistician or methodologist when setting up a trial. If no data are available to base a power calculation on, a number of assumptions need to be made using the concept of effect size [56,57]. 

### 4.3. Randomization and Blinding

An important aspect of randomized controlled trials is blinding to prevent bias at any stage of a trial [58]. Blinding not only prevents bias but helps distinguish between the actual therapeutic and placebo effects induced by receiving treatment. Such an effect has been shown in DBS for PD [59,60,61] and is mainly mediated by the patient’s expectations and concerns for the outcome of the procedure. Another effect is the placebo effect, or a reduction of therapeutic benefit due to the uncertainty of being allocated to placebo [62,63]. 

When setting up a trial, it is important to include at least two groups that only differ in respect to the treatment they receive. In a surgical context, blinding can be controversial, especially when concerning neurosurgery [64]. In DBS, two options are available as control conditions: sham surgery and sham stimulation.

The optimal placebo should appear exactly like the “real” treatment but lack the “supposed” specific component [65]. When considering sham surgery, it can be labeled an invasive placebo control using a procedure mimicking surgery as closely as possible. Such a sham surgery may include anesthesia, making an incision, and drilling burr holes without further implantation of an electrode [66]. In contrast to placebo-controlled drug studies, there are risks associated with sham surgery. Because of these risks and the absence of an actual treatment, sham surgery is generally deemed unacceptable [62,67]. Therefore, trials using DBS are likely to use a design employing sham stimulation. Here, the control condition consists of individuals who have the device implanted, but it is not yet or not fully activated [44].

In the case of sham stimulation, both groups of participants are implanted with the DBS electrode and battery and only differ in the stimulation they receive. One factor to take into account in this situation is the potential placebo/nocebo effect of the surgery itself on the outcome measures. This effect should be equal in both groups since both go through surgery. This can be controlled for by also measuring the outcome measures after surgery, potentially right after the two-week recovery and before stimulation is turned on to account for the potential effects of the surgery itself. 

During sham stimulation, blinding of the stimulation condition is particularly challenging. It has been shown for several targeted brain areas that patients are instantaneously aware when the stimulation is switched on or off [61]. Often, the desired clinical effects rely on stimulating above the threshold of conscious awareness [43]. This is especially problematic when there is a period of individual programming to define the optimal DBS parameters prior to randomization. During this period, patients will become familiar with the sensation of the stimulation. Such concerns become even more important in trials concerning subjective outcome measures, such as pain or quality of life ratings, which are often used in psychiatry. 

One strategy to deal with the disadvantages of sham stimulation is adding a run-in phase to the sham stimulation. This is accomplished by shortly performing stimulation at the start of the sham period and decreasing the stimulation settings step-by-step, creating the illusion of stimulation ON and the accompanying habituation to the sensation. This lowers the risk of unblinding [62].

Another approach is to use a crossover design in which all patients are implanted with electrodes and are split into two groups that go through both conditions in the opposite order. Using a crossover design is especially helpful in these early trials since they require fewer participants to achieve significance [68]. However, a blinding check should be performed at the start and end of each period in order to ensure that blinding was successful. It is possible to combine a crossover design with a run-in period in the sham condition. When using a crossover design, one should take into account that effects could carry over to the other condition, and therefore, a washout period is advisable. Additionally, one should check the effects of the order of treatment. The order of stimulation (ON–OFF and OFF–ON) could lead to different effects since experiencing the stimulation ON first could affect the way a patient judges the stimulation OFF period. 

### 4.4. Choosing Stimulation Parameters

Several characteristics of stimulation can be adjusted, such as the duration (pulse width in ms), amplitude (in mV or mA), and frequency (Hz), as well as the stimulation field [14]. Fine-tuning of the stimulation parameters should be adequately performed for every individual patient and can take several months. Optimal settings may depend on the disorder that is being treated, the targeted symptoms, and the neuroanatomical location [69]. It is advised to search the literature for common settings that are beneficial for the investigated indication. When unavailable, it is best to choose general parameters and adjust them in a predefined manner. Additionally, information from preclinical research on the effects of high- or low-frequency stimulation on the investigated disorder could be used to determine a starting point. 

An important advantage of DBS is that the clinical effect is reversible. Therefore, both therapeutic and side effects can be controlled by adjusting the stimulation parameters [26,70]. We suggest starting the adjustment of the settings not earlier than two weeks after surgery to avoid bias from the temporary lesion effect by surrounding edema around the electrode. Adjustments should be conducted regularly, for example, weekly for a period of six weeks. The goal of these adjustments is to strike a balance between the beneficial effects of the stimulation and undesired side effects. It is expected that there is an optimal range in which stimulation for a specific disorder will be situated (e.g., high- or low-frequency stimulation). Within this range, the most beneficial settings are individually determined for each participant and may vary over time. The adjustments to reach the optimal stimulation paradigm may take longer than the suggested adjustment period. Therefore, there should be an option to extend the adjustment period. When dealing with indications that depend on subjective measures, the use of standardized and validated measures is of utmost importance. Lastly, blinding the DBS adjustment procedure and ensuring that all changes are performed uniformly and recorded in a serial manner is advised.

### 4.5. Choosing Primary and Secondary Outcome Measures

A pivotal goal of a phase 1 trial is providing evidence of the safety of DBS in the targeted brain area to treat a particular disorder. This is accomplished by reporting the effect (using a measure of disorder severity), observing side effects, and inducing optimal settings for each participant. Additionally, measuring quality of life, common comorbidities, and the impression of primary caregivers could also prove advantageous. Today, one should also consider that wearable sensors or momentary assessments using a mobile phone as clinical evaluation tools (questionnaires and clinical tests) are not designed for the continuous, naturalistic (real-world) symptom monitoring needed to optimize clinical therapy to treat symptom fluctuations. Implementing eHealth in the outcome measures should thus become common practice [71]. Lastly, a longer, open-label follow-up condition can be considered to evaluate the long-term effects of stimulation and potentially capture changes that were not immediately present.

For further outcome measures, a balance needs to be struck between the individual and scientific benefit and the burden for the patient. Because DBS studies provide insight into the pathophysiology of a disorder, gathering perioperative electrophysiological information, such as MER and LFP, can also provide clinically relevant information. 

### 4.6. Care after the Study

When a trial ends, it is typically not the last time one sees a patient. It is important to guarantee post-trial care to ensure patient safety and treatment continuation. Issues such as battery replacement and setting adjustments should be guaranteed and clarified to the patient during the informed consent procedure. When a trial is performed in a DBS center, this can be achieved by enrolling the patient in the standard post-DBS care program.

## 5. Concluding Remarks

There is a need for clear guidelines for first-in-human DBS studies. For this reason, we propose a design a process similar to the one currently used for testing new pharmaceutical therapies. This gives a clear framework to operate under when designing a first-in-human DBS trial that contributes to the coherence and comparability of studies within the field. 

In setting up a trial, several aspects are important (see Table 3). Firstly, it is essential to collect as much preclinical and clinical evidence as possible to make a compelling case in favor of feasibility and safety. Preferably, this information should come from both animal and human studies. Only then should a first-in-human trial be undertaken. Strict patient selection criteria and extensive informed consent procedures, as well as in-depth preoperative screenings, are essential to achieve a favorable risk–benefit ratio for each patient. All possible steps should be taken to minimize side effects and adverse events. With these recommendations, we strive to further the discussion of international guidelines for DBS trials to ensure reproducibility and safeguard patients.

## Figures and Tables

**Table 1:** Comparison between phases in drug trials and suggested phases in DBS trials.

	Pharmacotherapy	Deep Brain Stimulation (Existing Active Medical Device)
**Preclinical**	Discovery and early screening Animal testing to assess: Safety Side effects	Discovery of potential new indications and gathering of first evidence of potential benefits Animal testing to assess: Target Stimulation settings Safety Side effects
**Phase 1** In small group	Evaluate safety Determine safe dosage Identify side effects	Evaluate safety of selected target Determine therapeutic window of stimulation parameters Identify side effects and complications
**Phase 2** In small to medium group	Further evaluate safety Test effectiveness	Further evaluate safety Test effectiveness
**Phase 3** In medium to large group	Confirm effectiveness Monitor side effects Compare to other treatments	Confirm effectiveness Monitor side effects Compare to other treatments
**Phase 4** In broader population	Provide additional information after approval, including risks, benefits, and best use	Provide additional information after approval, including risks, benefits, and best use

**Table 2:** Advised guidelines for patient selection and inclusion.

Criteria	Background	Suggestion
**Presence of the disorder**	Certainty of the presence of the disorder	All other treatable causes should be ruled out
**Severe suffering**	Evaluation of suffering depends on indication	Set cut-off score on specific disorder-specific measurement
		Take daily life functioning into account
**Refractory to existing therapies**	All approved general procedures should have been applied and not have made a substantial improvement	Important to list all possible approved treatments and check whether the patient received them and what the gain was
		Try to provide or refer to treatments that were not tried
		Combine with daily life functioning and severity score for decision
**Comorbidities**	No serious somatic comorbidities and preferably no cognitive deficits	Consultations with multidisciplinary team (general physician, anesthesiologist, neurologists. and psychiatrist) are necessary
		A balance should be struck between the risks and benefits for each patient
**MRI**	MRI rules and regulations	Consider exclusion of patients with MRI contraindication and no previous information
**Neuropsychological evaluation**	No significant pre-existing neuropsychological abnormalities	Exclude patients with significant pre-existing neuropsychological abnormalities in comparison to what can be expected of the general patient population
		Should be evaluated by a psychiatrist
		If depression or anxiety pre-existed before the disorder onset, and are thus not part or a result of the target disorder, it is advised to exclude the patient
		If it was a consequence of the disorder, it can be counseled until manageable
		When so debilitating that it poses an additional risk for participation in the trial, the patient should be excluded
**Age**	Evaluation on an individual basis	No specific cut-off score for age should be used
		Consider patients’ abilities individually
**Willingness and ability to give informed consent**	Evaluation on an individual basis	Adhere to (inter)national ethics regulations and guidelines
		Personal circumstances should not significantly impact the outcome

**Table 3:** Summary of the advised guidelines for patient selection and inclusion.

Summary of the Suggested Guidelines
Trial registration - Registration in an international database
Etiology of the disorder - Reversible causes should be ruled out
Severity of the disorder - Use a predetermined cut-off score of a validated measure - Include daily life functioning
Refractory to existing therapies - No substantial improvement with all available treatments (medical and psychological), or debilitating side effects occurred
Comorbidities - Patient should not have comorbidities with a short life expectancy - Preferably no cognitive deficits (unless linked to the indication, such as dementia)
Multidisciplinary team - A multidisciplinary team, including a neurosurgeon, neurologists and/or psychiatrist, and an anesthesiologist. evaluates potential risks and benefits for each individual before inclusion
MRI - High-resolution MRI for targeting - Patients with a contraindication to undergo MRI should be excluded
Neuropsychological and psychiatric evaluation - Patients should not have significant pre-existing neuropsychological abnormalities (as expected within the patient population) - Exclude patients with depression or anxiety that existed prior to disorder onset - Counsel patients with depression or anxiety as a consequence of the disorder before considering inclusion - Exclude patients with psychiatric or neuropsychological comorbidities that pose an additional risk for participation
Age - No specific cut-off for age is advised
Power - Prove feasibility and effect in order to provide sufficient power in the second phase
Randomization and blinding - Using a sham condition closely mimicking the real condition - Preferably using sham stimulation over sham surgery - Using a crossover design because of the expected smaller sample size
Stimulation parameters - Start adjustments less than two weeks after surgery to avoid interference of temporary lesion effects - Stimulation parameters should be adjusted and optimized for each individual - Adjustment process may take weeks to months
Primary and secondary outcome measures - Primarily reporting on safety trough effect, surgical complication, stimulation-induced side effects, and stimulation parameters - Quality of life
Aftercare - Ensure proper long-term aftercare
